# Identification of a glutamine metabolism reprogramming signature for predicting prognosis, immunotherapy efficacy, and drug candidates in bladder cancer

**DOI:** 10.3389/fimmu.2023.1111319

**Published:** 2023-02-23

**Authors:** Yan Xu, Zhixiu Xia, Xiaoyu Sun, Baojun Wei, Yang Fu, Du Shi, Yuyan Zhu

**Affiliations:** ^1^ Department of Urology, The First Hospital of China Medical University, Shenyang, China; ^2^ Colorectal Tumor Surgery Ward, Department of General Surgery, Shengjing Hospital of China Medical University, Shenyang, China; ^3^ Department of Pharmacology, School of Pharmacy, China Medical University, Shenyang, China

**Keywords:** bladder cancer, glutamine metabolism, immunotherapy efficacy, prognosis, molecular docking

## Abstract

**Background:**

Bladder cancer is the most common malignancy of the urinary system. However, patient prognosis and treatment outcomes in bladder cancer are difficult to predict owing to high tumor heterogeneity. Given that abnormal glutamine metabolism has been identified as a key factor driving the progression of bladder cancer, it is necessary to assess the prognosis and therapeutic efficacy of bladder cancer treatments based on an analysis of glutamine metabolism-related genes.

**Methods:**

We used bladder cancer sample data downloaded from The Cancer Genome Atlas to identify glutamine metabolism-related genes as prognostic markers, and established a novel Glutamine Metabolism Immunity Index (GMII) based on univariate and multivariate COX regression analyses. On the basis of GMII values, bladder cancer patients were divided into high- and low-risk groups, and systematic analysis was conducted for clinical features, somatic mutations, immune cell infiltration, chemotherapeutic response, and immunotherapeutic efficacy. Candidate small-molecule drugs targeting the GMII core target proteins were identified based on molecular docking analysis.

**Results:**

The GMII consisting of eight independent prognostic genes was established to be an excellent tool for predicting the survival in patients with bladder cancer and was validated using multiple datasets. Compared with patients in the high-risk group, those in the low-risk group had significantly better responses to gemcitabine and immune checkpoint blockade. In addition, we predicted 12 potential small-molecule drugs that could bind to three of the GMII core target proteins.

**Conclusions:**

The GMII can be used to accurately predict the prognosis and immunotherapeutic response of bladder cancer patients, as well as candidate small-molecule drugs. Furthermore, the novel “Glutamine Metabolism-related Gene”-guided strategy for predicting survival and chemo-immunotherapeutic efficacy may also be applicable for cancers other than bladder cancer.

## Background

Bladder cancer is the most common malignancy affecting the urinary system, for which there is high recurrence and mortality rates worldwide (1, 2). Despite advances in surgical treatment, cisplatin-based chemotherapy, and immunotherapy for bladder cancer patients, the 5-year overall survival rate remains low, ranging from 23% to 48% (3). With continued bladder cancer progression, tumor cells require larger amounts of nutrients to sustain their growth, resulting in abnormal metabolism within the tumor microenvironment (4). In this regard. aberrant metabolic pathways have previously been identified as potentially effective biomarkers and therapeutic targets in cancer (5), and prognostic and therapeutic predictions based on such pathways can contribute to enhancing comprehensive individualized treatment outcomes.

Glutamine, which is considered the most abundant and versatile free amino acid (6). is bound by amino acid transporters, following which, it is enzymatically converted to glutamate by glutaminase. Glutamate is subsequently metabolized to α-ketoglutarate *via* glutamate dehydrogenase or transaminase, which then enters the tricarboxylic acid cycle (TCA) to replenish circulating metabolites (7). In many types of cancer, including bladder cancer, glutamine metabolism is dysregulated, which is integral to the rapid proliferation of most tumor cells (8). Given that the efficiency of glutamine import and metabolism is essential for cancer cell viability (9), glutamine is viewed as an attractive target for cancer antimetabolite therapy. Several proteins and enzymes are used as biomarkers to guide tumor diagnosis and treatment, and in this regard, the findings of recent studies have indicated that whereas the primary energy source for most cells is glucose, some immune cells metabolize glutamine at a higher rate under conditions of catabolic stress (10, 11). Glutamine serves as an important source of reduced nitrogen to fuel the synthesis of biomacromolecules, such as nucleotides, that are important for tumor cell proliferation, invasion, and immune escape. However, glutamine is also an essential metabolite for immune cell activation and antitumor effects in the tumor microenvironment (12). Consequently, it is also necessary to take into consideration glutamine metabolism from the perspective of tumor immunotherapy (13, 14). Although glucose metabolism in the context of bladder cancer has been widely studied, the role played by glutamine metabolism in this cancer type is still unclear. Consequently, it is necessary to assess the prognostic importance of glutamine metabolism in bladder cancer based on glutamine metabolism-related genes, and to predict which bladder cancer patient subtypes would respond better to immunotherapy and chemotherapy.

In this study, we molecularly subtyped bladder cancer patients based on glutamine metabolism-related genes and combined a range of statistical algorithms to construct a Glutamine Metabolism Immunity Index (GMII) comprising eight genes involved in glutamine metabolism. We used this GMII to predict tumor immune cell infiltration, chemotherapeutic response, and immunotherapeutic effect, and conducted comprehensive validations. In addition, on the basis of molecular docking analysis, we identified potential small-molecule drugs that bind effectively to glutamine metabolism core target proteins.

## Materials and methods

### Data sources and preprocessing

We from the cancer genome atlas (TCGA) database (https://portal.gdc.cancer.gov/) to download the bladder cancer patients (including 414 samples of bladder cancer and 19 adjacent non tumor samples) expression of the spectral data, clinical information, somatic mutation. From Gene Expression Omnibus (GEO) database (https://cancergenome.nih.gov/) download GSE13507 (n = 165) and GSE32894 (n = 224) as an independent verification of the queue. The list of genes involved in glutamine metabolism was obtained from the GenesCards database.

### Analysis of differentially expressed glutamine genes

The R package “limma” was used to perform the difference analysis with the cutoff value set to p value<0.05. Gene Ontology (GO) and Kyto Encyclopedia of Genes and Genomes (KEGGE) analyses were performed using R package clusterProfiler. The STRING database was used to analyze protein-protein interactions (PPI) (15), and Cytoscape was used to visualize PPI networks (16). We identified key modules by using the ‘MCODE’ plug-in in Cytoscape, and used seven algorithms commonly used in ‘cytohubba’ plug-in (Closeness, Degree, EPC, Radiality, Stress, MCC, MNC) to identify Hub genes. TRRUST databasewas used to predict the transcription factor (TF) of Hub gene (17).

### Identification of glutamine metabolism-associated clusters

Bladder cancer samples were clustered by R package “ConsensusClusterPlus” to identify molecular subtypes related to glutamine metabolism. The R package “Survival” was used to perform Kaplan-Meier (KM) survival curves to compare outcomes between the two clusters.

### Construction and validation of prognostic features from glutamine metabolism-related genes and their derived GMII

Univariate Cox regression analysis was used to screen out the genes associated with overall survival (OS) of bladder cancer, and multivariate Cox regression analysis was used to establish GMII. The Glutamine Metabolism Immunity Index (GMII) was calculated for each patient according to the following formula: Glutamine Metabolism Immunity Index (GMII) = Coef(Gene1) × Expr(Gene1) + Coef(Gene2) × Expr(Gene2) +…… Coef(Genen) × Expr(Genen). Expr(Genen) represents the expression level of a specific gene, and Coef(Genen) represents the coefficient in multivariate Cox analysis. The prognostic value of the features was verified by KM analysis and Receiver Operation Characteristic (ROC) curve, and the prognostic characteristics were verified by GSE13507. Univariate and multivariate Cox analyses were performed to determine whether the characteristics were independent risk factors. According to the clinical characteristic parameters, the correlation and stratification analysis between GMII and clinical characteristics were performed, and the nomogram was constructed to compare the consistency between predicted and actual survival rates by 1-year, 3-year and 5-year calibration maps.

### Pan-cancer analysis

The GSCALite platform was used to analyze the eight genes of GMII (18). Fourteen pairs of normal and tumor tissue samples were selected for differential expression analysis. The prognosis of eight GMII genes was analyzed in 33 pan-cancer cancers. Gene set variation analysis (GSVA) was used to score the expression of these eight genes in each pan-cancer sample, and the GSVA score was obtained. The GSVA score was used to analyze the expression, prognosis and immune cell infiltration of these genes in pan-cancer. We also performed mutational analyses of the eight genes, including single nucleotide variation, copy number variation and methylation. Through CTRP database, we analyzed the expression levels and drug sensitivity of eight genes of GMII.

### Immunohistochemical analysis

Build GMII gene immunohistochemical from Human Protein Atlas (HPA) database (https://www.proteinatlas.org/) (19).

### Gene set enrichment analysis

We performed GSEA analysis by R package “clusterProfiler” to evaluate the main enrichment pathways in high-GMII groups to explore the underlying biological mechanisms. Filter for | NES |> 1, nominal p value < 0.05. Sample replacement was tested 1000 times, and clustering analysis of enriched gene sets was performed using the R package “enrichplot”. Reference genomes include Hallmark, c5go, and c2kegg.

### Gene mutation analysis

We calculated the tumor mutation burden (TMB) for each patient from somatic mutation data and compared TMB between high-GMII and low-GMII groups. The waterfall map is depicted through the R package “Maftools”, showing the mutation landscape of the high-GMII and low-GMII groups. We also performed mutually exclusive and collaborative analyses of genes with the highest frequency of mutations in the high-GMII and low-GMII groups. Finally, somatic mutations of GMII genes were identified by cBioPortal database.

### Immunogenomic landscape analysis

CIBERSORT method used to quantify infiltrating immune cell ratio (https://cibersort.stanford.edu/) (20). The proportion of 22 immune cells (B-naive cells, B-cell memory, plasma cells, T-cell CD8, T-cell CD4 naive, T-cell follicular helper cells, T-cell CD4 memory resting, T-cell CD4 memory activation, regulatory T cells (Tregs), γδ cells, monocytes, activation) was calculated by CIBERSORT method NK cells, resting NK cells, macrophage M0, macrophage M1, macrophage M2, resting dendritic cells, activated dendritic cells, resting mast cells, activated mast cells, eosinophils, and neutrophils). Samples with P<0.05 indicated that the proportion of immune cells calculated by CIBERSORT was correct. The tumor purity, stromal score, immune score and ESTIMATE scorewere calculated for each tumor sample by R package “ESTIMATE” (21). A single sample Gene set enrichment analysis (ssGSEA) algorithm was used to assess the immune infiltration between the two groups based on 28 immune cell types. XCELL, QUANTISEQ, MCPCOUNTER, EPIC and CIBERSORT-ABS software were also used to quantify the relative proportion of immune cell infiltration.

### Analysis of chemotherapeutic drug sensitivity between different GMII groups

From tumor susceptibility multiple omics (GDSC) database (https://www.cancerrxgene.org/) (22), download the cancer gene expression data of different drugs, through the calculation of R packages “pRRophetic” IC50 to assess patient response to common chemotherapy drugs.

### Prediction of immunotherapy response

Tumor Immune Dysfunction and Exclusion (TIDE) algorithm was used to infer the clinical response of patients to immunotherapy (23). High TIDE scores were associated with poorer immunotherapy. In addition, we extracted the IMvigor210 dataset, a group of clinical information on the treatment of urothelial carcinoma by anti-PD-L1 monoclonal antibody (atezolizumab) (24). The relationship between bladder cancer anti-PD-1 and anti-CTLA4 by Immunophenoscores(IPS) scores and GMII. The IPS score is a predictive score for a patient’s response to anti-PD-1 and anti-ctLA-4 treatments (25). was downloaded from TCIA database (https://tcia.at/home). These results were used to evaluate the predictive value of GMII for immune checkpoint therapy.

### Molecular docking simulation

We used MOE software to screen FDA-approved drugs that bind to target proteins and perform molecular docking simulations. Protein structures of core targets were collected from the PDB database and FDA-approved drugs were collected from the zinc15 database and converted to 3D structures in MOE by energy minimization. We optimized the protonation state of the protein and the direction of hydrogen at the PH of LigX 7 and the temperature of 300K. Finally, we studied the binding mode of PPARG, SLC7A9 and GALK1 with small molecule drugs by rigid docking simulation.

### Statistical analysis

Survival curves were drawn using the Kaplan-Meier method. Wilcoxon test and Kruskal-Wallis test were used for comparison between two groups and more than two groups, respectively. Correlation was assessed by Spearman correlation analysis. A P value of 0.05 or less was considered statistically significant. All statistical analyses were performed by R (version 4.1.1).

## Results

### Identification of genes related to glutamine metabolism and their biological functions

**Figure 1:**
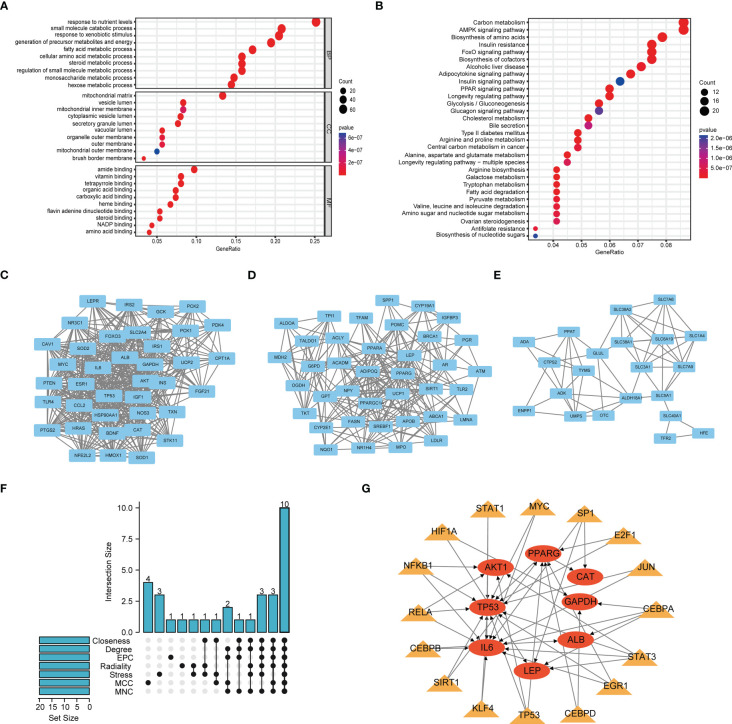
Function and subnetwork analysis of genes related to glutamine metabolism. **(A)** The top 10 rich terms for Biological Progress(BP), Cellular Component(CC) and Molecular Function(MF) in GO analysis. **(B)** The top 30 rich terms in KEGG’s analysis. **(C–E)** MCODE plug-in gets three main network modules. **(F)** UpSet of cytohubba plug-in seven algorithms. **(G)** Transcription factors predicted by TRRUST for Hub genes. Circles represent genes and triangles represent transcription factors.

The flow chart of this study is shown in **Figure S1**. We obtained 501 genes related to glutamine metabolism from the GeneCards database, and the screening criteria were that the correlation score was greater than 8 and they were protein-coding genes. Differential analysis of BC and normal bladder tissues yielded 301 genes. GO and KEGG analysis were performed to investigate the functions of these genes related to glutamine metabolism. The GO results showed that the genes related to glutamine metabolism were mainly enriched in the biological functions related to energy and metabolism. KEGG results showed that genes related to glutamine metabolism were enriched in signaling pathways such as carbon metabolism, AMPK signaling pathway and amino acid production (**Figures 1A, B**). PPI networks were analyzed by STRING database and visualized by Cytoscape to obtain three main network diagrams (**Figures 1C–E**). The common Hub genes were obtained by seven algorithms in Cytoscape software and the transcription factors of Hub genes were predicted in TRRUST database (**Figures 1F, G**).

### Identification of clusters related to glutamine metabolism and correlation analysis between clusters and immune microenvironment

**Figure 2:**
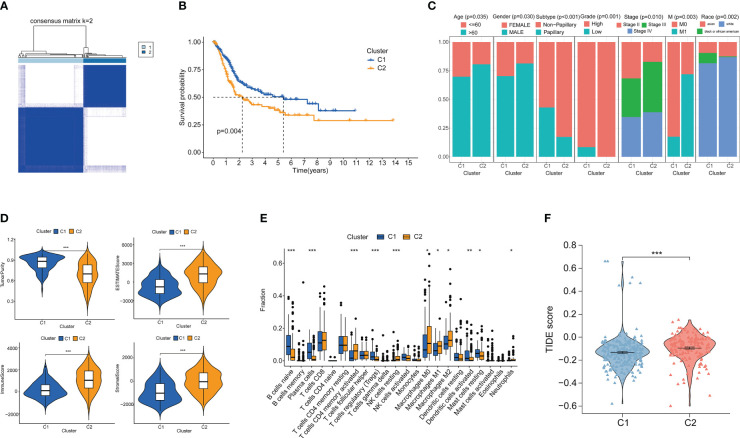
Cluster analysis and the association between different clusters with tumor microenvironments and immunotherapy. **(A)** Consensus clustering heat map when k=2. **(B)** Survival curves of two clusters. **(C)** Age, Gender, Subtype, Grade, Stage, M stage and Race between the two clusters. **(D)** Differences in tumor purity, ESITIMATE score, stroma and immune cells between the two clusters. **(E)** CIBERSORT score of immune cell infiltration between the two clusters. **(F)** TIDE score was used to compare the immunotherapy effect between Cluster 1 and Cluster 2. *p < 0.05 、**p < 0.01, ***p < 0.001.

Clustering analysis of bladder cancer patients with glutamine metabolism-related genes obtained after differential analysis showed that BC patients were best divided into two clusters, with good internal consistency and stability of each Cluster (**Figure 2A**). The general characteristics of Cluster 1 and Cluster 2 are shown in **Table S1**. The survival curve showed that Cluster2 had a poor prognosis (p < 0.05) (**Figure 2B**). There were significant differences in clinical parameters such as age, gender, subtype, grade, clinical stage, M and race of bladder cancer between the two clusters. Compared with patients with C1 bladder cancer, the proportions of patients older than 60, male, non-papillary invasive subtype, high grade, high stage, M1, white and Asian in patients with C2 bladder cancer were significantly higher than those in patients with C1 bladder cancer (p < 0.05) (**Figure 2C**).

We analyzed the immune microenvironment between the two clusters. Through ESITIMATE algorithm, according to the Cluster 2 has higher ESITIMATE score, immune score, score matrix and lower purity of tumor (**Figure 2D**). The CIBERSORT algorithm showed that Cluster 2 had higher immune cell infiltration (**Figure 2E**). Patients’ response to immune checkpoint inhibitors was negatively correlated with TIDE score. We found that TIDE score of Cluster 2 patients was significantly higher than that of Cluster 1 patients (p < 0.05), indicating that Cluster 2 patients had poor effect on immune checkpoint inhibitors (**Figure 2F**).

### Construction and validation of the GMII

**Figure 3:**
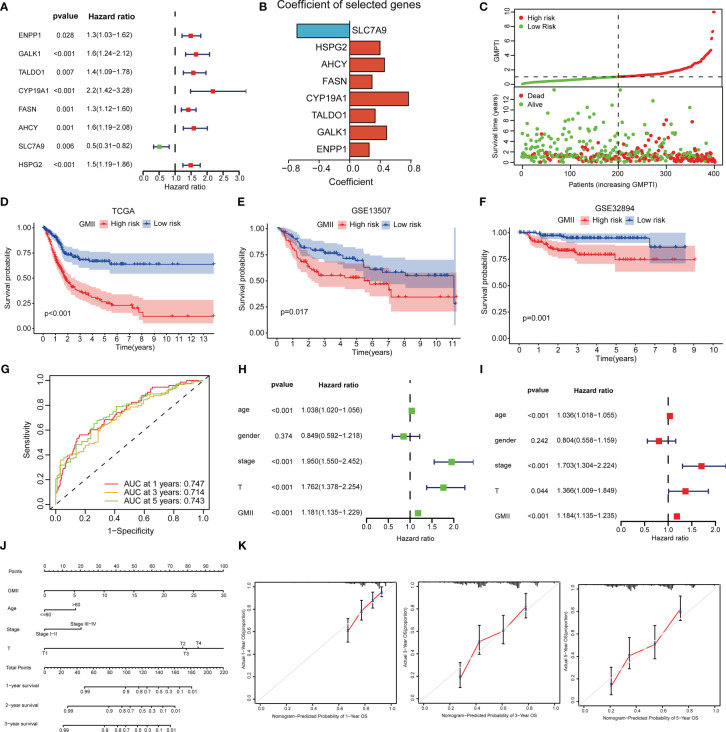
Construction of prognostic treatment index related to glutamine metabolism and validation of external data sets. **(A)** Forest maps of eight GRGs obtained by multivariate Cox analysis. **(B)** Construct eight GRGs coefficients of GMII. **(C)** Survival state of TCGA queue. **(D)** Kaplan-Meier survival curve of the TCGA cohort. **(E)** Kaplan-Meier survival curve of the GSE13507 cohort. **(F)** Kaplan-Meier survival curve of the GSE32894 cohort. **(G)** ROC curve of TCGA queue. **(H, I)** univariate and multivariate regression analysis. **(J)** Histogram based on GMII, age, clinical stage, and T-stage. **(K)** Calibration curves show the consistency of 1-year, 3-year, and 5-year survival rates predicted from bias-adjusted prognostic columns with actual survival rates.

To construct a risk model associated with glutamine metabolism and its derived GMII, eight genes with independent prognostic value were identified by univariate and multivariate Cox analyses to construct GMII (**Figure 3A**). Coefficients for each gene in GMII (**Figure 3B**). Correlation analysis between GMII and survival status showed that higher GMII was associated with higher mortality (**Figure 3C**). Patients in the high-GMII group had a worse prognosis than those in the low-GMII group (p < 0.05) (**Figure 3D**). To verify the predictive value of this GMII, GSE13507 and GSE32894 were used to verify that the mortality rate in the high-GMII group was significantly higher than that in the low-GMII group (p < 0.05) (**Figures 3E, F**). The area under the 1-year, 3-year and 5-year ROC curves were 0.747, 0.714 and 0.743, respectively (**Figure 3G**), suggesting that GMII could better predict the short-term and long-term survival status of bladder cancer patients. The general characteristics of TCGA, GSE13507 and GSE32894 patients are shown in **Table S2**. Univariate and multivariate regression analyses showed that GMII was an independent risk factor (**Figures 3H, I**). Based on the results of multivariate analysis, a nomogram was constructed based on GMII, age, clinical stage, and T stage, with GMII accounting for the majority of the total score (**Figure 3J**). Calibration curves showed that the predicted and actual 1 -, 3 -, and 5-year survival rates were consistent with the reference lines (**Figure 3K**).

The expression of five of the eight GMII genes (TALDO1, AHCY, FASN, GALK1 and SLC7A9) in bladder cancer tissues was higher than that in normal tissues, while ENPP1, CYP19A1 and HSPG2 were down-regulated in tumor tissues (p < 0.05) (**Figure S2A**). Immunohistochemical data of HPA showed that FASN showed moderate staining in the cytoplasm and nucleus of urothelial cancer cells, but no staining was detected in normal bladder tissues. HSPG2 showed high staining in the cytoplasm and nucleus of normal bladder cells, but no staining was detected in urothelial carcinoma cells (**Figure S2B**). Glutamine Related Genes (GRGs) is highly expressed in most tumor tissues and is a high risk factor (**Figures S3A, B**). Gene set variation analysis (GSVA) was performed on 8 GRGs, and GSVA score was positively correlated with the expression of representative gene sets. Most tumor tissues had significantly higher GSVA scores than normal tissues (**Figure S3C**). GSVA score was significantly correlated with the prognosis of bladder cancer (p < 0.05), including overall survival (OS), progression-free survival (PFS) and disease-free survival (DSS) (**Figure S3D**). We further evaluated the correlation between GSVA score and immune cell infiltration and showed that GSVA score was significantly correlated with immune cell infiltration in bladder cancer (p < 0.05) (**Figure S3E**). We also collected the features of published prognostic models for bladder cancer and compared the GMII features with their prognostic prediction accuracy. The results showed that GMII values were superior to other models in terms of prognostic prediction (**Figure S4**).

### Clinical correlations analysis of GMII

**Figure 4:**
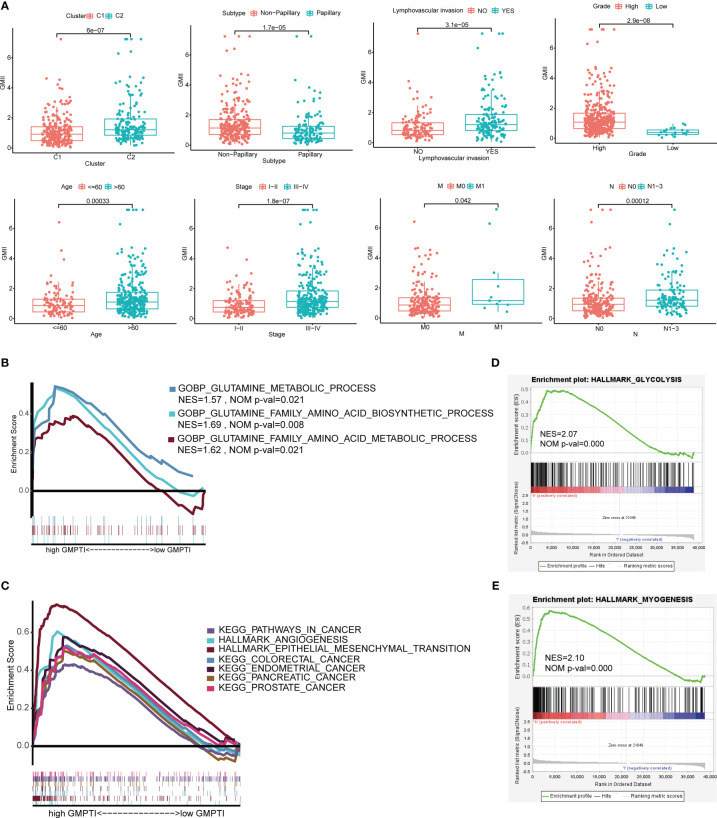
Correlation between GMII and clinical traits and gene set enrichment analysis in high-GMII group. **(A)** Differences in GMII between different clinical feature groups. **(B)** Pathways related to glutamine metabolism are abundant in the high-GMII group. **(C)** Glycolysis was significantly enriched in the high-GMII group. **(D)** Pathways associated with tumor development and progression are abundant in the high-GMII group. **(E)** Muscle synthesis was significantly enriched in the high-GMII group.

To further verify the clinical significance of GMII, we analyzed the differences in GMII between different clinical characteristics groups. The results showed that patients with Cluster 2, Non-papillary infiltration, lymphovascular invasion, High Grade, stage III-IV, M1 and N1-3 had higher GMII, suggesting that the higher the GMII, the more advanced the tumor (**Figure 4A**). Stratified analysis showed that GMII could significantly differentiate the prognosis of almost all clinical subgroups, with patients in the high-GMII group having a worse prognosis (**Figure S5**). To investigate the pathways that regulate tumorigenesis in the high-GMII group, we performed GSEA analysis, and the results showed that, The high GMII group was significantly enriched in high glutamine metabolism, glutamine synthesis and decomposition (p < 0.05) (**Figure 4B**). In addition, the high-GMII group was significantly enriched in carcinogenesis, angiogenesis, and epithelial-mesenchymal transition (EMT) pathways (p < 0.05) (**Figure 4C**). Glycolysis and myosynthesis were the features with the highest NES in the high-GMII group (**Figures 4D, E**).

### Relationship between GMII and immune microenvironment

**Figure 5:**
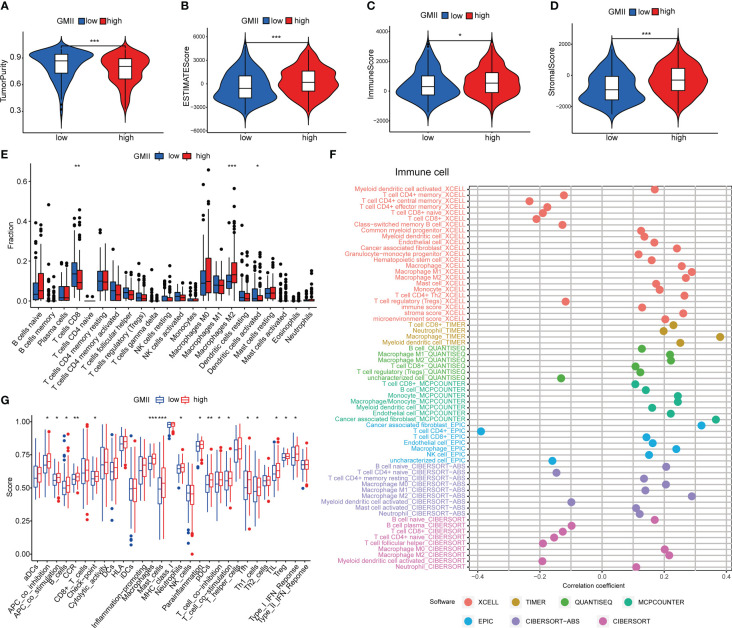
Immune cell infiltration in high-GMII and low-GMII groups. Tumor purity **(A)**, ESTIMATE score **(B)**, immune score **(C)**, and stromal score **(D)** between high – and low-GMII populations. **(E)** CIBERSORT algorithm was used to analyze the contents of immune cell infiltration in high-GMII and low-GMII groups. **(F)** Different software evaluated the correlation between immune scores and immune cells. **(G)** ssGSEA algorithm showed immune cell infiltration of immune-related functions and pathways in high-GMII and low-GMII groups. *p < 0.05 、**p < 0.01, ***p < 0.001.

Previous studies have shown that glutamine metabolism and tumor microenvironment play an important role in tumor development (7, 26). The ESTIMATE algorithm found that the high GMII group had lower tumor purity (**Figure 5A**) and higher ESTIMATE score (**Figure 5B**), immune score (**Figure 5C**) and stromal score (**Figure 5D**). CIBERSORT algorithm showed that the level of CD8 T cells in the high-GMII group was significantly lower than that in the low-GMII group (p < 0.05), and the level of immunosuppressive M2-type macrophages was increased in the high-GMII group, suggesting that the high-GMII group had higher immunosuppressive activity and promoted tumor progression (**Figure 5E**). We found correlations between GMII and a variety of immune cells using different software (**Figure 5F**). The ssGSEA algorithm results showed that the high-GMII group had higher immune cell infiltration and immune-related functions and pathways than the low-GMII group (**Figure 5G**).

### Relationship between GMII and somatic cell mutation

**Figure 6:**
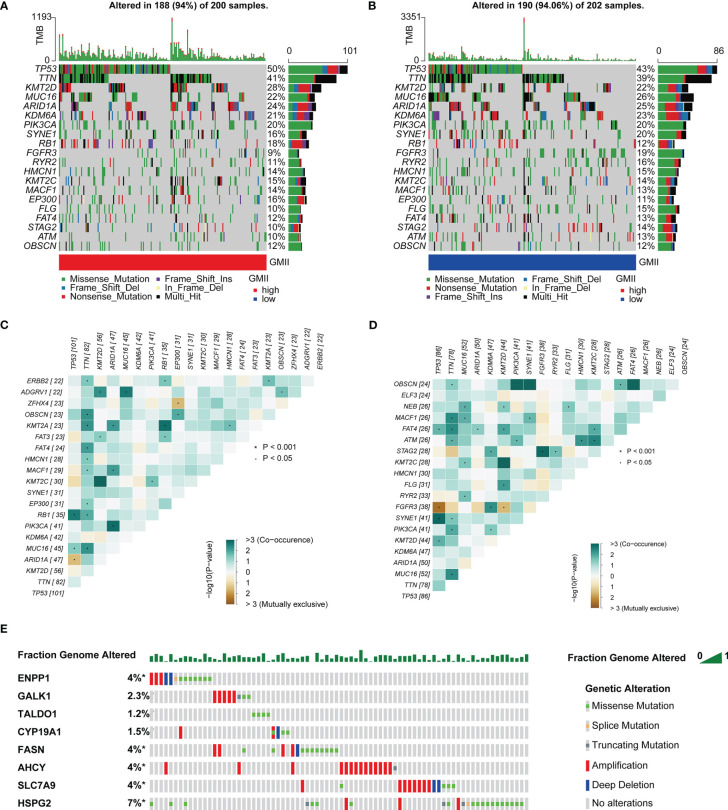
Association of GMII with genetic changes. Waterfall diagrams of somatic mutations in high-GMII **(A)** and low-GMII **(B)** groups. Mutual exclusion and synergistic heat maps of mutated genes in high-GMII **(C)** and low-GMII **(D)** groups. **(E)** Mutation rates of the eight GRGs that constructed GMII.

TMB is an important predictor of immunotherapy and chemotherapy. To further investigate the association of GMII with somatic mutations in cancer cells, we used single nucleotide variation data to investigate differences in genomic mutations between high-GMII and low-GMII groups. TP53, TTN, KMT2D, MUC16 and ARID1A were the top five genes with the highest mutation frequency in high-GMII and low-GMII populations, but the mutation frequency of each gene was different between the two groups (**Figures 6A, B**). Through mutual exclusion and cooperation analysis among mutated genes, it was found that there was gene mutation synergy between most genes, and significant mutational mutual exclusion of TP53-ARID1A and EP300-ZFHX4 was found in the high-GMII group (**Figure 6C**). Significant mutational mutual exclusion of TP53-FGFR3 and KMT2D-FGFR3 was also found in the low-GMII group (**Figure 6D**). There were no differences in TMB between the two groups or in survival curves between the high and low TMB groups (**Figure S6A, B**). After TMB combined with GMII, the prognosis of the high TMB+ low GMII group was significantly better than that of the low TMB+ high GMII group (**Figure S6C**). In addition, the mutation rate of eight genes in GMII was detected in the cBioPortal database, and it was found that the mutation rate was low (**Figure 6E**).

### Prediction of chemotherapy effects by the GMII

**Figure 7:**
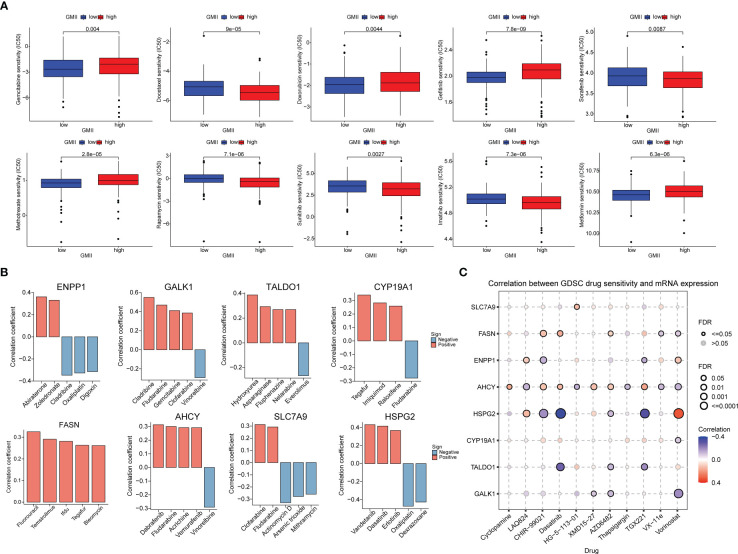
Effect of GMII on drug sensitivity. **(A)** Differences in response to commonly used chemotherapy drugs between high-GMII and low-GMII groups. **(B)** Construct the correlation between GMII gene and drug sensitivity. **(C)** Correlation between GDSC drug sensitivity and mRNA expression levels of eight genes that construct GMII.

To investigate the potential of GMII for predicting the response to chemotherapytic therapy, We first downloaded data from the GDSC database on the response of high-GMII and low-GMII populations to common chemotherapy agents. The results showed that many common bladder cancer chemotherapy drugs had significant differences among high-GMII groups (p<0.05). Gemcitabine, as the most common chemotherapy drugs for bladder cancer, had significantly lower IC50 values in the low-GMII group than in the high-GMII group (p<0.05), suggesting that Gemcitabine may have better efficacy in the low-GMII group (**Figure 7A**). We also mapped the 3D structures of chemotherapeutic agents with differences between the two groups using the PubChem database (**Figure S7**). The relationship between GRGs and drug sensitivity was analyzed from cellMiner database, and the histograms of the top five drugs with the highest correlation between genes and drug sensitivity were drawn. Positive correlation indicates that stronger gene expression is more sensitive to drugs, while negative correlation indicates that stronger gene expression is more resistant to drugs. Among them, up-regulation of FASN expression may lead to enhanced sensitivity of patients to most drugs (**Figure 7B**). In addition, GDSC database was used to analyze the relationship between drug sensitivity and mRNA expression of the eight GRGs used to construct GMII. Contrary to cellMiner database, positive correlation represented that gene expression was related to drug resistance, while negative correlation represented that gene expression was related to drug sensitivity. The relationship between AHCY gene expression and chemotherapy-drug sensitivity is extensive, and HSPG2 has the highest correlation with most chemotherapy-drug sensitivity, among which HSPG2 is correlated with Dasatinib sensitivity in both databases (**Figure 7C**). These results suggest that the expression changes of genes constructing GMII may be effective indicators for predicting drug response and as potential therapeutic targets.

### Prediction of immunotherapy efficacy by the GMII

**Figure 8:**
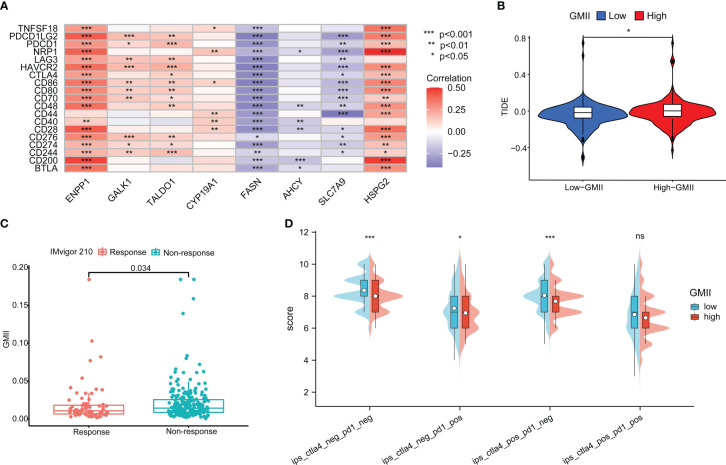
Application of GMII in immunotherapy prediction. **(A)** GMII gene expression was associated with common immune checkpoints. **(B)** TIDE scores were used to compare immunotherapy efficacy between high-GMII and low-GMII groups. **(C)** The IMvigor 210 database analyzed GMII in the responding and non-responding groups to immunotherapy. **(D)** The difference in response between high-GMII and low-GMII groups to PD1- or no-CTLA4 blockers, PD1 blockers, CTLA4 blockers, and PD1-CTLA4 co-blockers. *p < 0.05 、**p < 0.01, ***p < 0.001. ns, Non Significance.

Immune checkpoint inhibitors have provided clinical benefits in the treatment of a variety of tumors. By analyzing the correlation between genes in GMII and common immune checkpoints, we found that genes with the highest correlation with glutamine metabolism were significantly negatively correlated with PD-1/PD-L1 expression. For example, SLC7A9, a glutamine transporter, and FASN, a key enzyme in the metabolism of glutamine into fatty acids. These results indicated an inverse correlation between glutamine metabolism and PD-1/PD-L1 expression (**Figure 8A**). The expression of most immune checkpoints was different between the high-GMII group and the low-GMII group (p < 0.05) (**Figure S8**). Higher TIDE scores were associated with poorer immune checkpoint blockade therapy and shorter survival, and higher TIDE scores in the high-GMII group suggested poorer response to immune checkpoint blockade therapy (**Figure 8B**). The results of IMvigor210 dataset showed that there was a significant difference in GMII between the immunotherapy response group and the non-response group (p<0.05), and the GMII was lower in the response group (**Figure 8C**). The scores of IPS, IPS-PD1 blocker, IPS-CTLA4 blocker, and IPS-PD1-CTLA4 co-blocker were lower in the high-GMII group, indicating that the high-GMII group had a poor effect of anti-PD1, anti-CTLA4, and anti-PD1-CTLA4 co-treatment (**Figure 8D**). These results suggest that GMII may be associated with immunotherapy in bladder cancer patients.

### Identification of core target proteins and prediction of drug candidates

**Figure 9:**
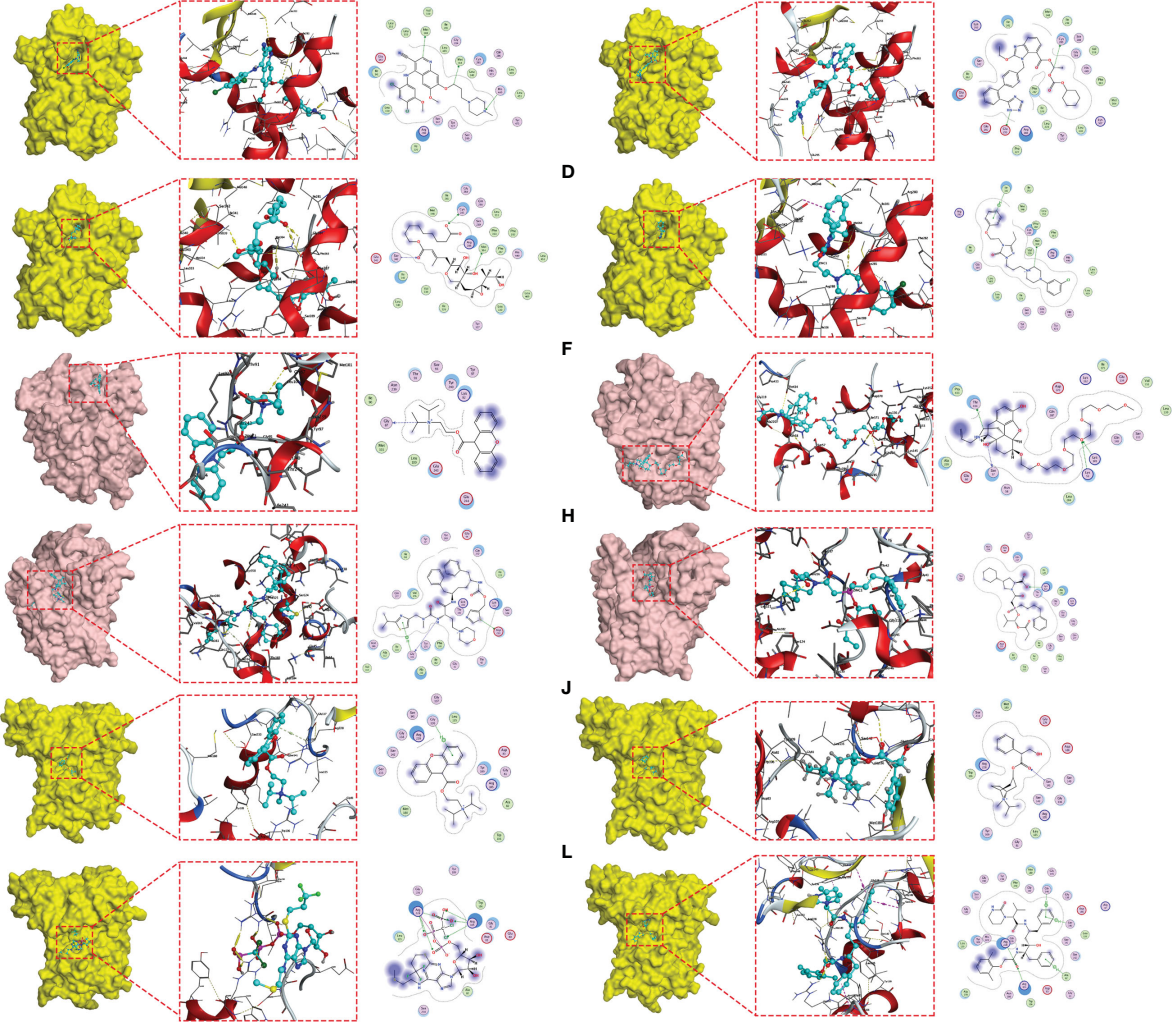
Molecular docking posture. Candidate drugs and target proteins screened using molecular docking. The Figure shows the docking position of the PPARG active pocket with Bosulif **(A)**, Candesartan **(B)**, Centany **(C)** and Nefazodone **(D)**. Docking position of the SLC7A9 active pocket with Propantheline **(E)**, Naloxegol **(F)**, Cobicistat **(G)** and Fosinopril **(H)**. Docking position of GALK1 active pocket with Propantheline **(I)**, Ipratropium **(J)**, Cangrelor **(K)** and Lopinavir **(L)**.

In order to identify the core targets of genes related to glutamine metabolism, we constructed a PPI network through the STRING database (confidence score greater than 0.4), found the most closely related subnetwork to the GMII gene and found the core gene PPARG by “MCODE” in Cytoscape software (**Figure S9**). PPARG is located at the hub of the network and has the highest degree among all nodes. SLC7A9 is potentially used as a glutamine transporter and GAPK1 as a targeted receptor with small molecule inhibitors of kinases. We obtained X-ray structures of PPARG, SLC7A9 and GALK1 from the PDB database to screen 1379 FDA-approved drugs potentially targeting PPARG, SLC7A9 and GALK1. We used rigid docking in MOE to simulate the binding mode of small molecule drugs to PPARG, SLC7A9 and GALK1, where the interaction relationship between target proteins and candidate small molecules is shown in **Table S3**. We show the top four small-molecule drugs with the highest binding ability to PPARG (Bosulif, Candesartan, Centany, and Nefazodone) (**Figures 9A–D**) and the top four small-molecule drugs with the highest binding ability to SLC7A9 (Propantheline, Naloxeg) ol, Cobicistat and Fosinopril) (**Figures 9E–H**) and the top four small-molecule drugs with the highest GALK1 binding capacity (Propantheline, Ipratropium, Cangrelor and Lopinavir) (**Figures 9I–L**). For example, Bosulif (ZINC000022448983) forms hydrogen bonds with PPARG amino acid residues Met-348, Met-364 and His-449, among which Met-348 and Met-364 act as hydrogen bond acceptor and His-449 act as hydrogen bond donor. Naloxegol (ZINC000095564694) forms hydrogen bonds with SLC7A9 non-transmembrane amino acid residues Lys-53, Ser-57, Lys-145 and Thr-434, among which Ser-57 and Thr-434 act as hydrogen bond acceptor. Lys-53 and Lys-145 act as hydrogen bond donors. Cangrelor (ZINC000085537017) formed hydrogen bond and ion bond related interaction with GAPK1 amino acid residues Arg-228 and Arg-105, among which Arg-228 and Arg-105 were hydrogen bond donors. In addition, these small molecules form van der Waals (VDW) interactions with residues around the protein receptor, which contribute to the binding energy between small molecules and PPARG.

## Discussion

Abnormal glutamine metabolism is considered a key factor driving the progression of solid tumors such as bladder cancer (8) (27); and consequently, there is an urgent need to identify more reliable and accurate glutamine metabolism-related markers that can be used to predict bladder cancer patient survival and immunotherapeutic response. In this study, we established the GMII consisting of eight glutamine metabolism-related genes (*ENPP1*, *GALK1*, *TALDO1*, *CYP19A1*, *FASN*, *AHCY*, *SLC7A9*, and *HSPG2*), a high value of which is associated with poor prognosis in bladder cancer patients. For comparative purposes, we evaluated the efficacy of other prognostic models for bladder cancer, which showed that the predictive performance of GMII was superior to that of all other assessed models. Furthermore, the GMII could be used to differentiate among patients with different levels of immune checkpoint expression, and predict their therapeutic response to ICI therapy. Moreover, on the basis of in silico molecular docking analysis, we identified potential drugs that can modulate the core target proteins of glutamine metabolism. Thus, aberrant glutamine metabolism signaling, as a reliable predictor of bladder cancer prognosis and immunotherapeutic response, may provide valuable insights for establishing effective therapeutic approaches for the treatment of bladder cancer.

In our previous study, we attempted to predict the prognosis of bladder cancer patients and their treatment response based on overall metabolic profiles (28). However, despite accumulating evidence that tumor-specific metabolic phenotypes are closely associated with prognosis and treatment response, there has to date been an insufficient assessment of gene signature indices focusing on key amino acid metabolic pathways, such as that involved in glutamine metabolism (13). In this study, we found that the GMII value is inversely associated with most immune checkpoint genes. We established that the TIDE score based on the high-GMII group (high GMII value) was significantly higher than that of the low-GMII group, indicating that ICI therapy is less effective for the treatment of patients with a high-GMII score. IPS has been established to be a better predictor of immunotherapy response in cancer patients undergoing anti-PD-1 and anti-CTLA-4 treatment (25). and we found that levels of IPS, IPS-PD1, IPS-CTLA4, and IPS-PD1-CTLA4 co-blockers were lower in the high-GMII group, thereby indicating that anti-PD-1 and anti-CTLA-4 treatments were less effective in the treatment of high-GMII group patients. Consistent with our observations, among patients in the IMvigor210 cohort with urothelial carcinoma, significant differences in GMII have been identified in the responders and non-responders to PD-L1 therapy. In the present study, we assessed the responses of bladder cancer patients to common chemotherapeutic drugs based on their IC50 values and calculated the association between gene expression and drug sensitivity, which revealed significant differences in the responses to common chemotherapeutic drugs such as gemcitabine in the two GMII groups. These findings thus indicate that the risk model we constructed and the derived GMII can serve as effective indicators for assessing the response of bladder cancer patients to chemotherapy and immunotherapy, and may provide useful guidance for the future treatment of these patients.

**Figure 10:**
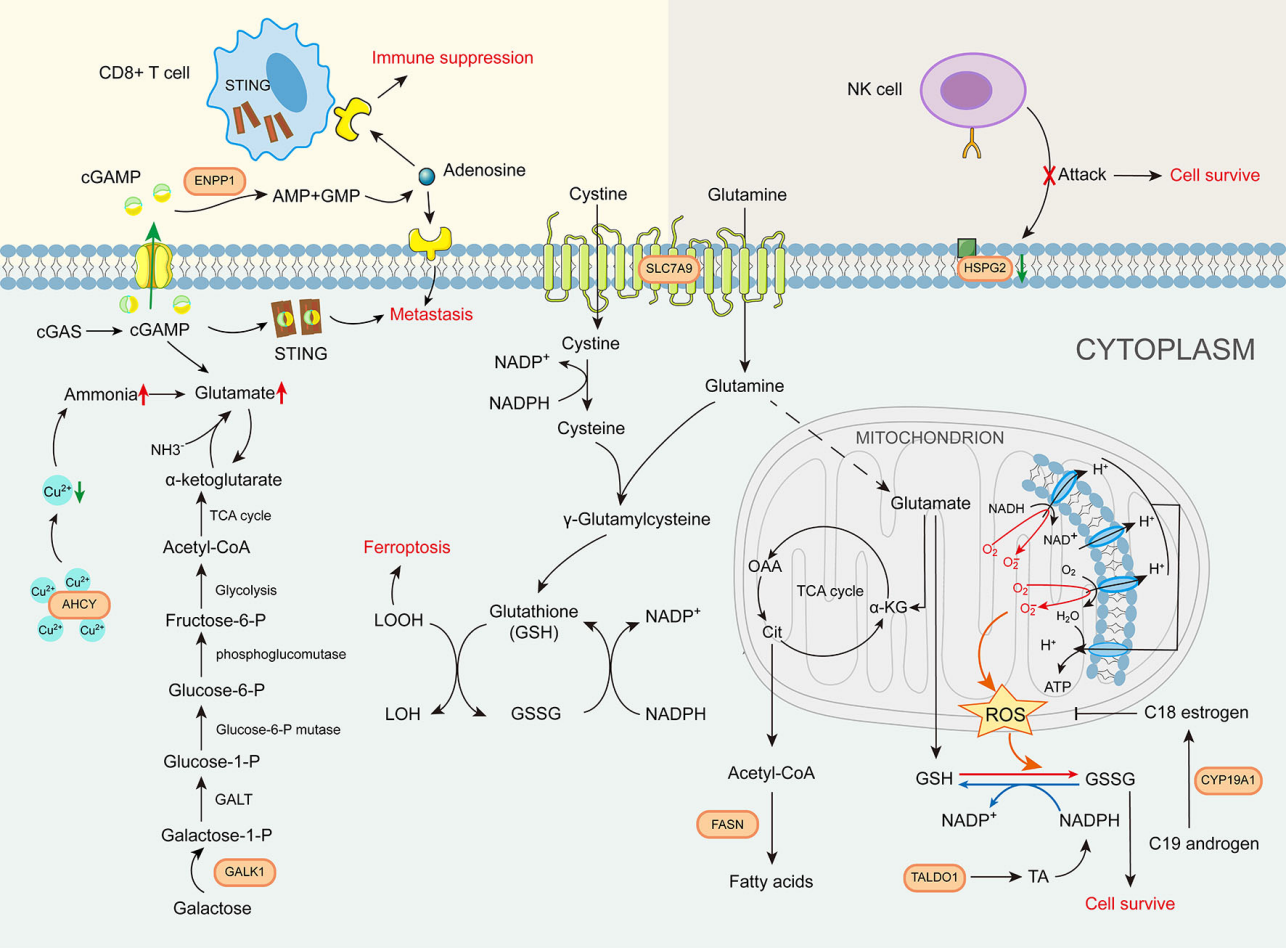
The role of glutamine metabolism immunity index gene in glutamine metabolism-related pathways.

The roles of the eight GMII genes in glutamine metabolism-related pathways can be characterized as follows. (1) Amino acid transport: Unlike SLC1A5, which is currently widely targeted in clinical studies, our analysis indicates that SLC7A9 is important for bladder cancer (29). Multiple studies have shown that SLC7A9 plays an essential role as a transporter of different amino acids for tumors, as these compete with immune cells for nutrients (30, 31). (2) Glutamate production: AHCY has a strong copper-binding capacity and is highly expressed, resulting in elevated glutamine levels (32, 33). GALK1 is a key enzyme in the catabolism of galactose, which can be further converted to glutamate by other enzymes (34). (3) Glutamate metabolism: FASN is a central regulator of *de novo* fatty acid synthesis that promotes the anabolic biosynthesis of fatty acids from citrate (7). *TALDO1* encodes a transaldolase (TA) that promotes nucleotide synthesis and the metabolic scavenging of ROS (35). and CYP19A1 is a cytochrome P450 (CYP) enzyme with immunomodulatory effects (36). (4) (4) Immune cell-related metabolism: ENPP1 promotes tumor cell metastasis and tumor immune escape (37). and HSPG2 is a cell-surface antigen that regulates NK cell activation (**Figure 10**). Given that the expression patterns of the aforementioned eight glutamine metabolic genes associated with tumorigenesis and regulation of the tumor immune microenvironment, the glutamine metabolism-related risk model and its derived GMII can be considered to closely reflect the prognosis of bladder cancer and predict the effects of chemotherapy and immunotherapy.

Our systematic study of immunological and chemotherapeutic differences between low- and high-GMII groups has provided important insights into the mechanisms underlying aberrant glutamine metabolism in bladder cancer and the predictive utility of the GMII. In this regard, the findings of several previous studies have revealed the key roles of glutamine metabolism and the tumor immune microenvironment in tumor progression (12). Bladder cancer cells have been established to utilize immune checkpoint molecules such as PD-L1 to induce immune escape, thereby generating an immunosuppressive tumor microenvironment around bladder cancer cells (38),. In addition, bladder cancer cells have been shown to induce the activation of tumor-associated macrophages and regulatory T (Treg) cells, which in turn suppresses the antitumor activity of CD8+ cytotoxic T cells (39, 40). By constructing the GMII for glutamine metabolism-related genes to identify their response to immunotherapy, we found that patients in the low-GMII group (low glutamine metabolism) showed a more significant response to PD-L1 and PD-1 blockade treatment. Correspondingly, we found that although patients in the high-GMII group (high glutamine metabolism) were characterized by higher immune infiltration, they also harbored a larger number immunosuppressive cells, such as Treg cells and M2 subtype macrophages, which are known to suppress anti-tumor CD8+ T cells. Indeed, we detected a significant reduction in the proportion of CD8+ T cells among patients in the high-GMII group. Overall, this further highlights the fact that specific immune microenvironments promote the progression of bladder cancer and govern the responses to immunotherapy. Consistent with this scenario, the findings of a recent study have revealed that by activating the EGFR/ERK/c-Jun pathway, glutamine deprivation can promote the upregulation of PD-L1 in bladder cancer cells (8). Moreover, blocking glutamine can induce different metabolic processes to overcome the immune escape of tumors and enhance the efficacy of immunotherapy (41, 42). These observations thus provide evidence to indicate a complex dynamic regulatory relationship between glutamine metabolism and the tumor immune microenvironment (43). Accordingly, the characterization of glutamine metabolism may represent a novel approach for screening treatment-receptive patients and enhancing the efficacy of immunotherapy. Conversely, however, the findings of previous studies have indicated that glutamine metabolism can provide raw materials for the over-activated glycolysis and oxidative phosphorylation of tumor cells, and by promoting metabolic homeostasis, can also contribute to inducing tumor cell resistance to chemotherapeutic drugs (44–46). The perturbation of glutamine metabolism has also been shown to enhance sensitivity to gemcitabine in different types of solid tumors (47). On the basis of the aforementioned observations, we further analyzed differences between the different glutamine metabolism groups with respect to the efficacy of responses to common chemotherapeutic drugs, and found that patients in the low-GMII group were characterized by a more pronounced sensitivity to common chemotherapeutic drugs such as gemcitabine. These findings thus indicate that characterizing glutamine metabolism can also serve as a reasonable and effective method for screening receptive patients and enhancing the efficacy of chemotherapy. Consequently, targeting glutamine metabolism combined with PD-1/PD-L1 checkpoint blockade therapy and/or chemotherapy could represent a potentially effective therapeutic strategy for improving treatment outcomes among bladder cancer patients.

As a further application of GMII efficacy prediction, we demonstrated the feasibility of combining core target and structure-based approaches to identify drug candidates. On the basis of a PPI network constructed using genes associated with glutamine metabolism, we identified PPARG as the major hub gene, and by employing molecular docking software, we used PPARG, SLC7A9, and GALK1 as small-molecule drug targets to screen for potential drugs from among those in the FDA-approved drug library. We accordingly found that the first four small molecules with the highest PPARG binding ability (Bosulif, Cadesartan, Centany and Nefazodone) bind to the NR-LBD domain of PPARG protein and prevent it from binding to the nuclear ligand. The top four small molecules with the highest GALK1 binding fraction (Propantheline, Ipratropium, Cangrelor and Lopinavir) bind to the ATP-binding site of GALK1, blocking ATP occupation and thus affecting the protein kinase function of GALK1. PPARG acts as a nuclear receptor that regulates multiple biological functions, including adipogenesis, metabolism, and immunity (48). and PPARG signaling has been reported to have an important influence on immune rejection in patients with bladder cancer (49). Among the four small molecule drugs with the highest affinity for PPARG, bosulif has been reported in clinical trials for chronic myeloid leukemia (50). In addition, propantheline, which can be used to enhance the efficacy of antiretroviral drugs, can target SLC7A9, which in turn affects the amino acid nutrition of the tumor microenvironment and thus tumor cell survival. Cangrelor, which has been shown to be beneficial for intraoperative antiplatelet therapy, and lopinacir, which has been used in the treatment of severe COVID-19, were found to have high affinity for GALK1 (51, 52). Although the specific mechanisms of action of these small-molecule compounds remain to be further investigated, our findings indicate that they have potential utility for tumor immunotherapy, particularly among bladder cancer patients with abnormal glutamine metabolism.

Despite our important findings, this study does have certain limitations. Notably, our analysis, and hence conclusions, are based on data obtained from public databases, which may accordingly have led to inherent case selection bias. In addition, although our findings were validated based on assessments using multiple external datasets, the evaluation of a larger number of clinical cases is necessary to further verify the accuracy of our results. Finally, further *in vivo* and *in vitro* experiments are needed to examine the function of GRGs in bladder cancer.

## Conclusion

In conclusion, based on the in-depth analysis of multiple aspects of bladder cancer based on the risk model and its derived GMII, we found that GMII can better predict the prognosis and immunotherapy response in BC patients. This study provides useful clues for the discovery of novel prognostic and therapeutic biomarkers and small-molecule drug targets from the perspective of oncogenic amino acid metabolic reprogramming. In an era when immunotherapy offers great promise for various cancer treatments, GMII provides guidance for the clinical diagnosis and individualized comprehensive treatment of bladder cancer.

## Data availability statement

The original contributions presented in the study are included in the article/**Supplementary Material**. Further inquiries can be directed to the corresponding authors.

## Author contributions

YX: formal analysis, data curation, conceptualization, writing original draft. ZX: formal analysis, visualization. XS: software, investigation. BW: investigation. YF: investigation. DS: software, supervision. YZ: conceptualization,writing—review and editing, supervision, project administration, funding acquisition. All authors contributed to the article and approved the submitted version.

## Supplementary material

Supplementary Table 1 Clinical statistical analysis of Cluster 1 and Cluster 2.

Supplementary Table 2 General characteristics of patients with TCGA, GSE13507 and GSE32894.

Supplementary Table 3 Interaction between target protein and candidate small molecules

Supplementary Figure 1 Flow chart of this study

Supplementary Figure 2 Immunohistochemistry analysis of FASN and HSPG2. **(A)** Differential expression of eight GMII-building GRGs between tumor tissue and normal tissue in the TCGA cohort. **(B)** Immunohistochemical results of FASN and HSPG2 in tumor and normal tissues. * *p* < 0.05, ***p* < 0.01, ****p* < 0.001.

Supplementary Figure 3 Pan-cancer analysis. **(A, B)** Differential expression levels and prognostic analysis of genes between tumor and normal tissues. **(C, D)** Difference level of GSVA scores and prognostic analysis between normal and tumor tissues. **(E)** The relationship between GRGs expression and immune cell infiltration. Red means positive correlation, blue means negative correlation. * *p* < 0.05, ***p* < 0.01, ****p* < 0.001, #FDR<0.05.

Supplementary Figure 4 Comparison of GMII index with other prognostic models of bladder cancer

Supplementary Figure 5 Prognosis curve among clinical subgroups

Supplementary Figure 6 Analysis of tumor mutation load. **(A)** TMB differences between high-GMII and low-GMII groups. **(B)** Survival curves of high-TMB and low-TMB groups. **(C)** Survival curve after combination of TMB and GMII.

Supplementary Figure 7 3D structures of chemotherapeutic drugs with differences between the two groups.

Supplementary Figure 8 Differences in the expression of common immune checkpoints between high-GMII and low-GMII groups.

Supplementary Figure 9 Subnetwork and core gene PPARG most closely related to GMII gene.

## Glossary

**Table:**

TCGA	The Cancer Genome Atlas
GEO	Gene Expression Omnibus
BC	Bladder Cancer
GRGs	Glutamine related Genes
GMII	Glutamine Metabolism Immunity Index
ENPP1	Ectonucleotide pyrophosphatase/phosphodiesterase 1
GALK1	Galactokinase 1
TALDO1	Transaldolase 1
CYP19A1	Cytochrome P450 Family 19 Subfamily A Member 1
FASN	Fatty Acid Synthase
AHCY	Adenosylhomocysteinase
SLC7A9	Solute Carrier Family 7 Member 9
HSPG2	Heparan Sulfate Proteoglycan 2
PD-1	Programmed cell death 1
PD-L1	Programmed cell death 1 ligand 1
CTLA4	Cytotoxic T-lymphocyte-associated protein 4
ICB	Immune checkpoint blockade
OS	Overall survival
ROC	Receiver Operation Characteristic
NES	Normalized enrichment score
TMB	Tumor mutation burden
IC50	half maximal inhibitory concentration
GDSC	Genomics of Drug Sensitivity in Cancer
PPI	protein–protein interaction
KM	Kaplan–Meier
ssGSEA	single-sample gene set enrichment analysis
TIDE	tumor immune dysfunction and exclusion
GSEA	gene set enrichment analysis
GO	Gene Ontology
KEGG	Kyoto Encyclopedia of Genes and Genomes
LAG3	Lymphocyte Activating 3
IDO1	Indoleamine 2, 3-Dioxygenase 1
PFS	Progression-free survival
DSS	Disease-free survival
TP53	Tumor Protein P53
TTN	Titin
KMT2D	Lysine Methyltransferase 2D
MUC16	Mucin 16
ARID1A	AT-Rich Interaction Domain 1A
EP300	E1A Binding Protein P300
ZFHX4	Zinc Finger Homeobox 4
FGFR3	Fibroblast Growth Factor Receptor 3
ADCC	Antibody-dependent cytotoxicity
ROS	Reactive oxygen species
GPX4	glutathione peroxidase 4
GSH	reduced glutathione
TCA	tricarboxylic acid
