# Supplementary material for: Identification of a glutamine metabolism reprogramming signature for predicting prognosis, immunotherapy efficacy, and drug candidates in bladder cancer
Source: Front Immunol. 2023 Feb 23;14:1111319. doi: 10.3389/fimmu.2023.1111319 (PMC9995899; doi:10.3389/fimmu.2023.1111319)
Supplement: Supplementary Table 3 — Interaction between target protein and candidate small molecules [file Table_3.docx]

**PPARG**

**A： 1I7I: ZINC000022448983（Bosulif）**

**Ligand Receptor Interaction Distance E (kcal/mol)**

**C15 15 SD MET 364 (A) H-donor 3.76 -0.5**

**C28 28 SD MET 348 (A) H-donor 4.13 -0.5**

**N21 21 NE2 HIS 449 (A) H-acceptor 3.02 -3.1**

**B：1I7I: ZINC000004074875（Cadesartan）**

**Ligand Receptor Interaction Distance E (kcal/mol)**

**C14 14 SG CYS 285 (A) H-donor 3.82 -1.1**

**N44 44 OE2 GLU 295 (A) H-donor 3.00 -8.6**

**C：1I7I: ZINC000004102194（Centany）**

**Ligand Receptor Interaction Distance E (kcal/mol)**

**O17 17 SG CYS 285 (A) H-donor 3.14 -0.6**

**O40 40 SD MET 364 (A) H-donor 3.43 -1.5**

**O5 5 N SER 342 (A) H-acceptor 2.98 -0.8**

**O17 17 SG CYS 285 (A) H-acceptor 3.14 -0.6**

**D：1I7I: ZINC000000538065（Nefazodone）**

**Ligand Receptor Interaction Distance E (kcal/mol)**

**C22 22 SD MET 364 (A) H-donor 4.15 -1.0**

**6-ring CD1 ILE 341 (A) pi-H 4.02 -0.5**

**SLC7A9**

**E：6YV1: ZINC000001530761（Propantheline）**

**Ligand Receptor Interaction Distance E (kcal/mol)**

**C25 25 O GLY 87 (A) H-donor 3.20 -0.6**

**F：6YV1: ZINC000095564694（Naloxegol）**

**Ligand Receptor Interaction Distance E (kcal/mol)**

**C6 6 OG1 THR 434 (A) H-donor 3.23 -0.5**

**O47 47 O SER 57 (A) H-donor 3.25 -1.1**

**O33 33 NZ LYS 53 (A) H-acceptor 3.18 -4.3**

**O33 33 NZ LYS 145 (A) H-acceptor 3.49 -0.5**

**G：6YV1: ZINC000085537014（Cobicistat）**

**Ligand Receptor Interaction Distance E (kcal/mol)**

**N12 12 O GLY 315 (A) H-donor 3.26 -2.0**

**S49 49 OD2 ASP 233 (A) H-donor 3.28 -1.6**

**5-ring CA GLY 315 (A) pi-H 4.74 -1.0**

**H：6YV1: ZINC000003977764（Fosinopril）**

**Ligand Receptor Interaction Distance E (kcal/mol)**

**O39 39 CE LYS 121 (A) H-acceptor 3.22 -1.2**

**GALK1**

**I：1WUU: ZINC000001530761（Propantheline）**

**Ligand Receptor Interaction Distance E (kcal/mol)**

**6-ring CA GLY 136 (A) pi-H 3.86 -0.8**

**J：1WUU: ZINC000100015775（Ipratropium）**

**Ligand Receptor Interaction Distance E (kcal/mol)**

**O17 17 N SER 141 (A) H-acceptor 3.17 -0.5**

**K：1WUU: ZINC000085537017（Cangrelor）**

**Ligand Receptor Interaction Distance E (kcal/mol)**

**O19 19 NH1 ARG 228 (A) H-acceptor 2.60 -4.9**

**O24 24 NH1 ARG 228 (A) H-acceptor 2.66 -2.6**

**CL27 27 NH2 ARG 105 (A) H-acceptor 2.84 -0.5**

**O30 30 NH2 ARG 105 (A) H-acceptor 2.84 -4.6**

**O30 30 NH2 ARG 228 (A) H-acceptor 3.29 -3.8**

**O31 31 NH2 ARG 105 (A) H-acceptor 2.96 -1.8**

**O19 19 NH1 ARG 228 (A) Ionic 2.60 -7.8**

**O20 20 NH1 ARG 228 (A) Ionic 3.05 -4.2**

**O24 24 NH1 ARG 228 (A) Ionic 2.66 -7.2**

**O24 24 NH2 ARG 228 (A) Ionic 3.68 -1.3**

**O30 30 NH2 ARG 105 (A) Ionic 2.84 -5.6**

**O30 30 NH2 ARG 228 (A) Ionic 3.29 -2.8**

**L：1WUU: ZINC000003951740（Lopinavir）**

**Ligand Receptor Interaction Distance E (kcal/mol)**

**O12 12 NH2 ARG 105 (A) H-acceptor 2.95 -1.2**

**O12 12 NH2 ARG 228 (A) H-acceptor 2.83 -2.0**

**6-ring CA ALA 82 (A) pi-H 4.34 -0.5**

**6-ring CA SER 140 (A) pi-H 4.08 -0.7**

**6-ring N GLY 346 (A) pi-H 4.89 -0.6**
