# Supplementary figures and images for: Identification of a glutamine metabolism reprogramming signature for predicting prognosis, immunotherapy efficacy, and drug candidates in bladder cancer
Source: Front Immunol. 2023 Feb 23;14:1111319. doi: 10.3389/fimmu.2023.1111319 (PMC9995899; doi:10.3389/fimmu.2023.1111319)

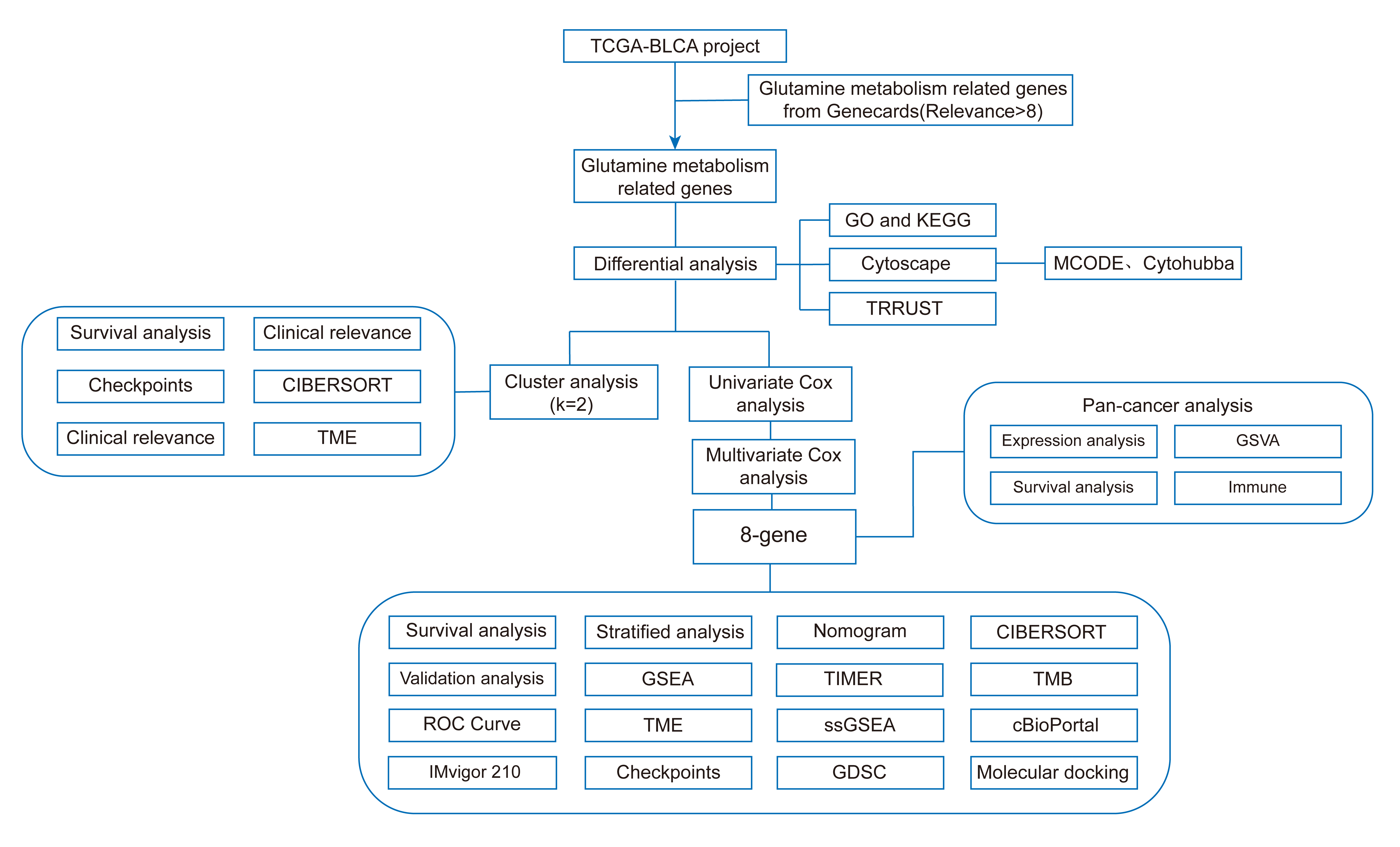

Supplement: Supplementary Figure 1 — Flow chart of this study [file Image_1.tif]

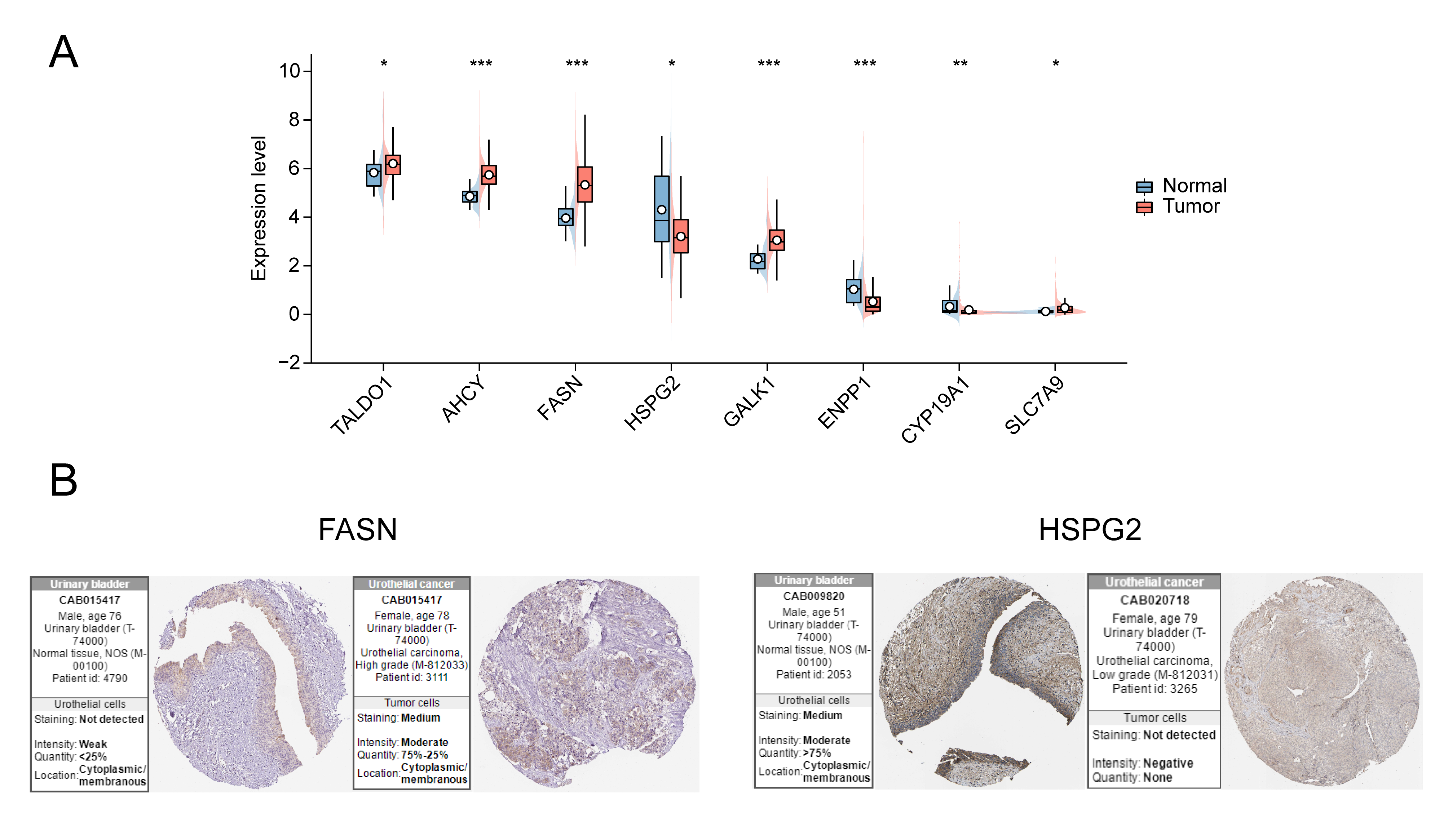

Supplement: Supplementary Figure 2 — Immunohistochemistry analysis of FASN and HSPG2. (A) Differential expression of eight GMII-building GRGs between tumor tissue and normal tissue in the TCGA cohort. (B) Immunohistochemical results of FASN and HSPG2 in tumor and normal tissues. * p < 0.05, **p < 0.01, ***p < 0.001. [file Image_2.tif]

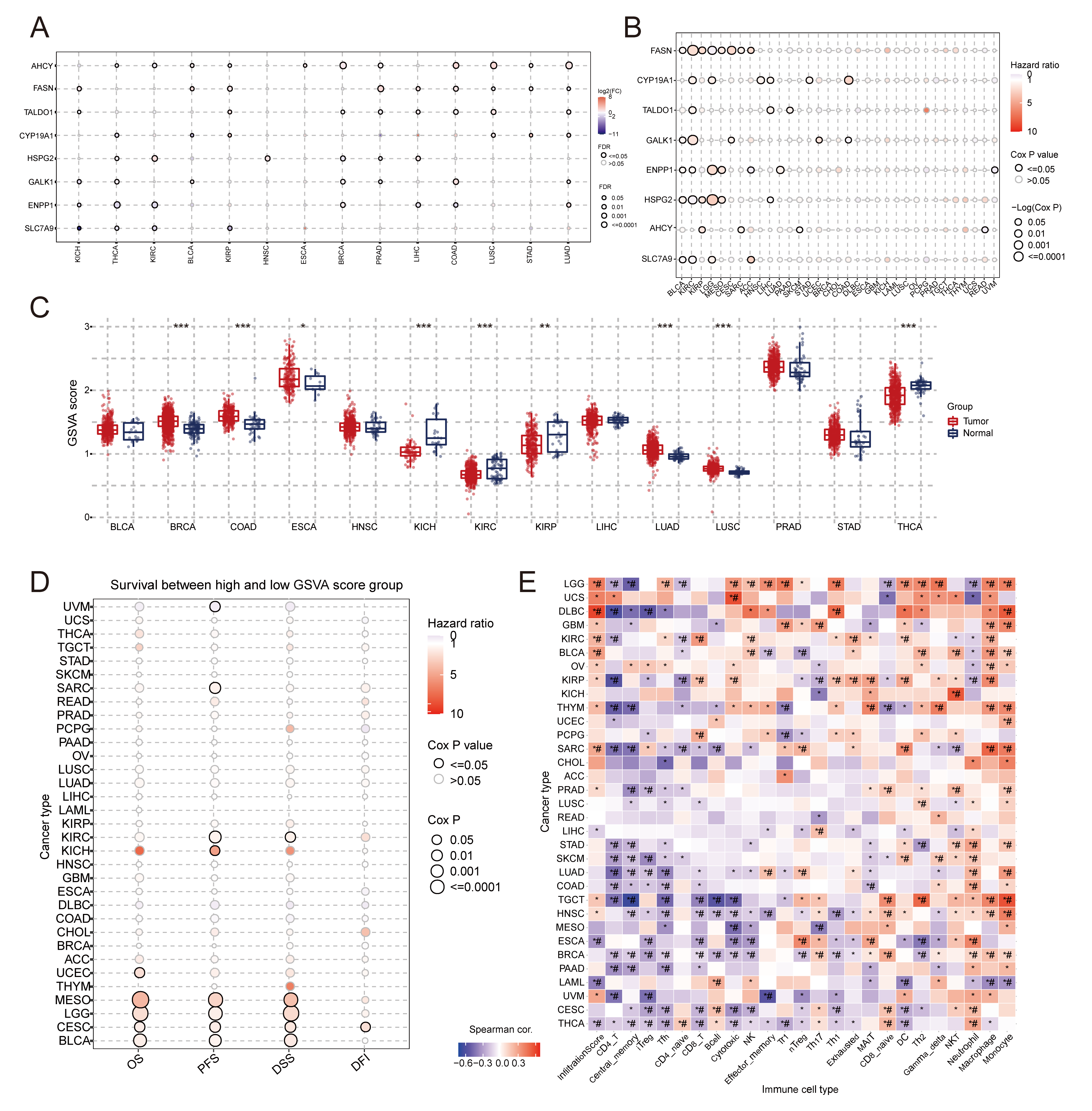

Supplement: Supplementary Figure 3 — Pan-cancer analysis. (A, B) Differential expression levels and prognostic analysis of genes between tumor and normal tissues. (C, D) Difference level of GSVA scores and prognostic analysis between normal and tumor tissues. (E) The relationship between GRGs expression and immune cell infiltration. Red means positive correlation, blue means negative correlation. * p < 0.05, **p < 0.01, ***p < 0.001, #FDR<0.05. [file Image_3.tif]

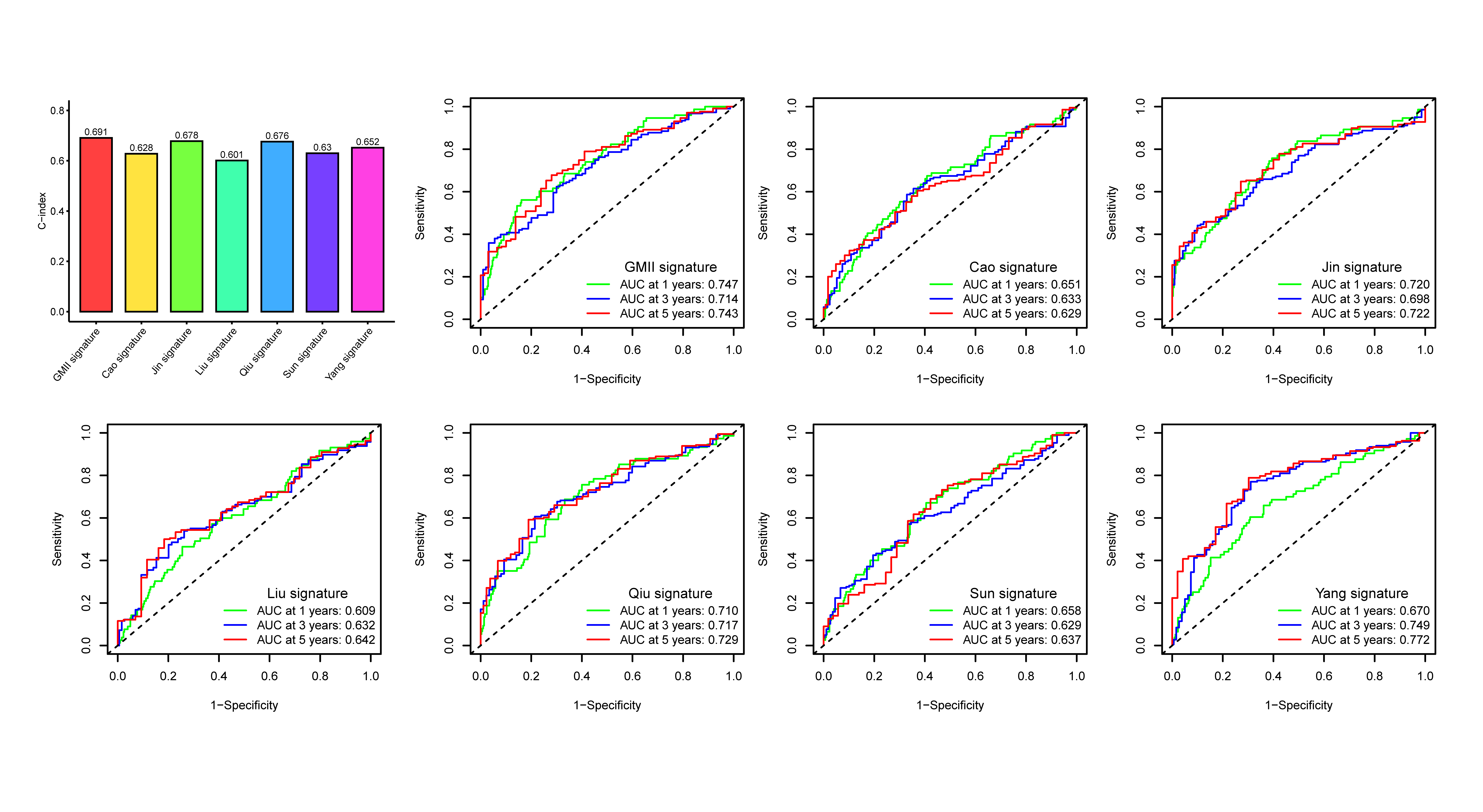

Supplement: Supplementary Figure 4 — Comparison of GMII index with other prognostic models of bladder cancer [file Image_4.tif]

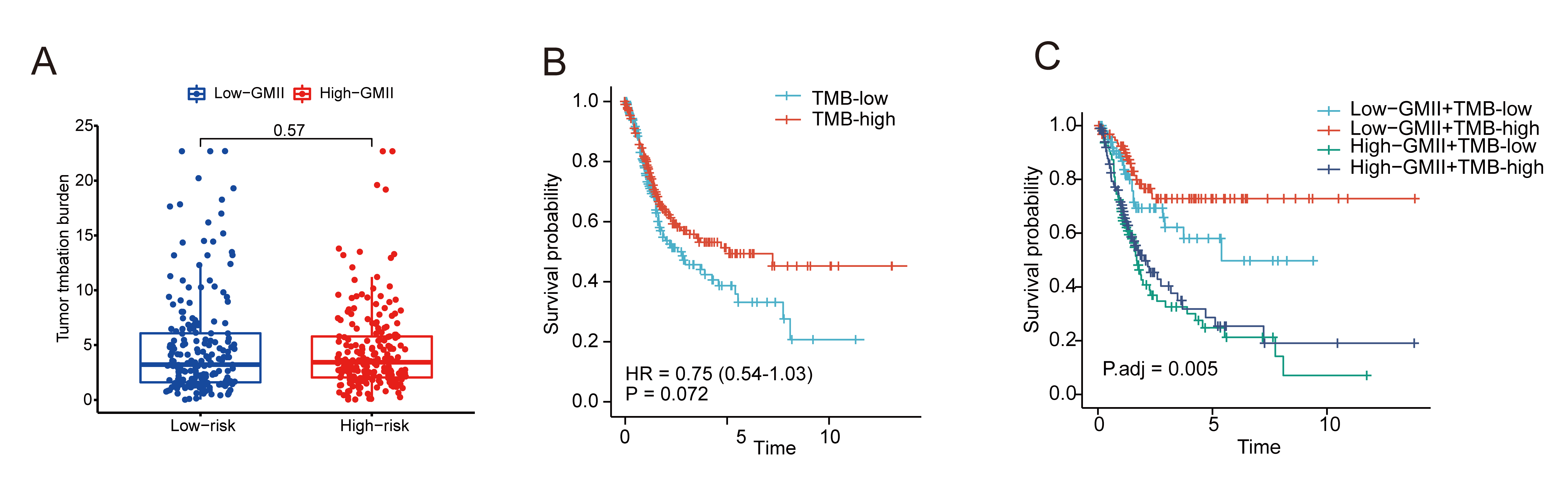

Supplement: Supplementary Figure 6 — Analysis of tumor mutation load. (A) TMB differences between high-GMII and low-GMII groups. (B) Survival curves of high-TMB and low-TMB groups. (C) Survival curve after combination of TMB and GMII. [file Image_6.tif]

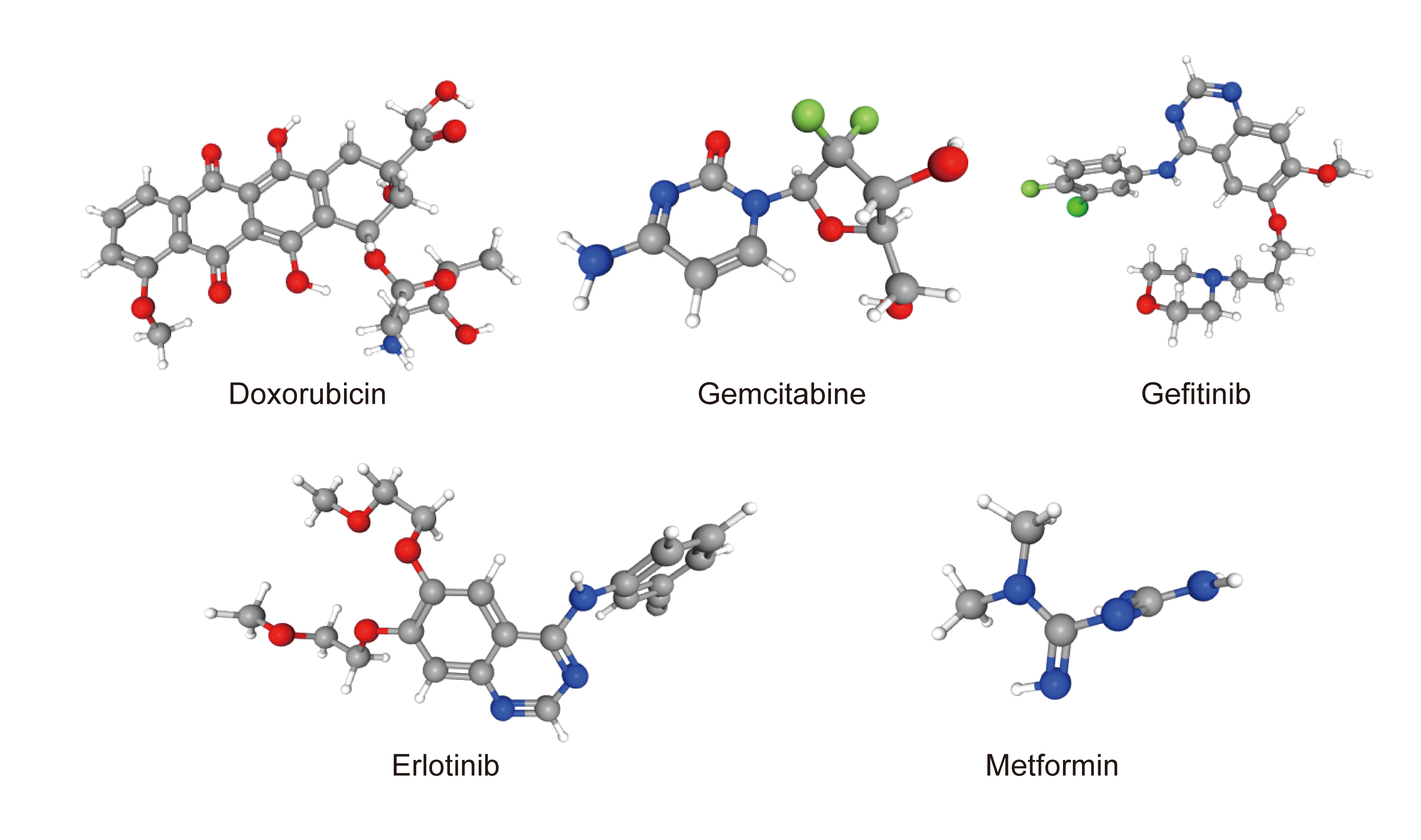

Supplement: Supplementary Figure 7 — 3D structures of chemotherapeutic drugs with differences between the two groups. [file Image_7.tif]

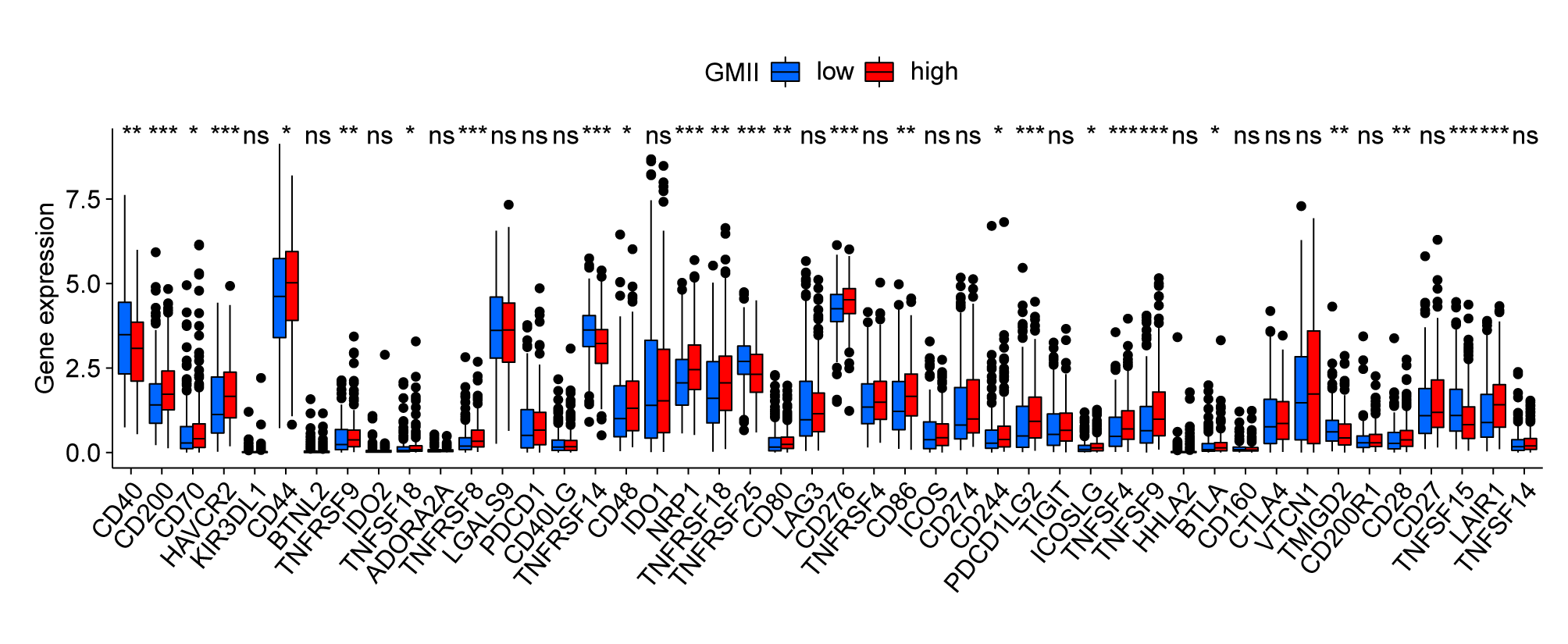

Supplement: Supplementary Figure 8 — Differences in the expression of common immune checkpoints between high-GMII and low-GMII groups. [file Image_8.tif]

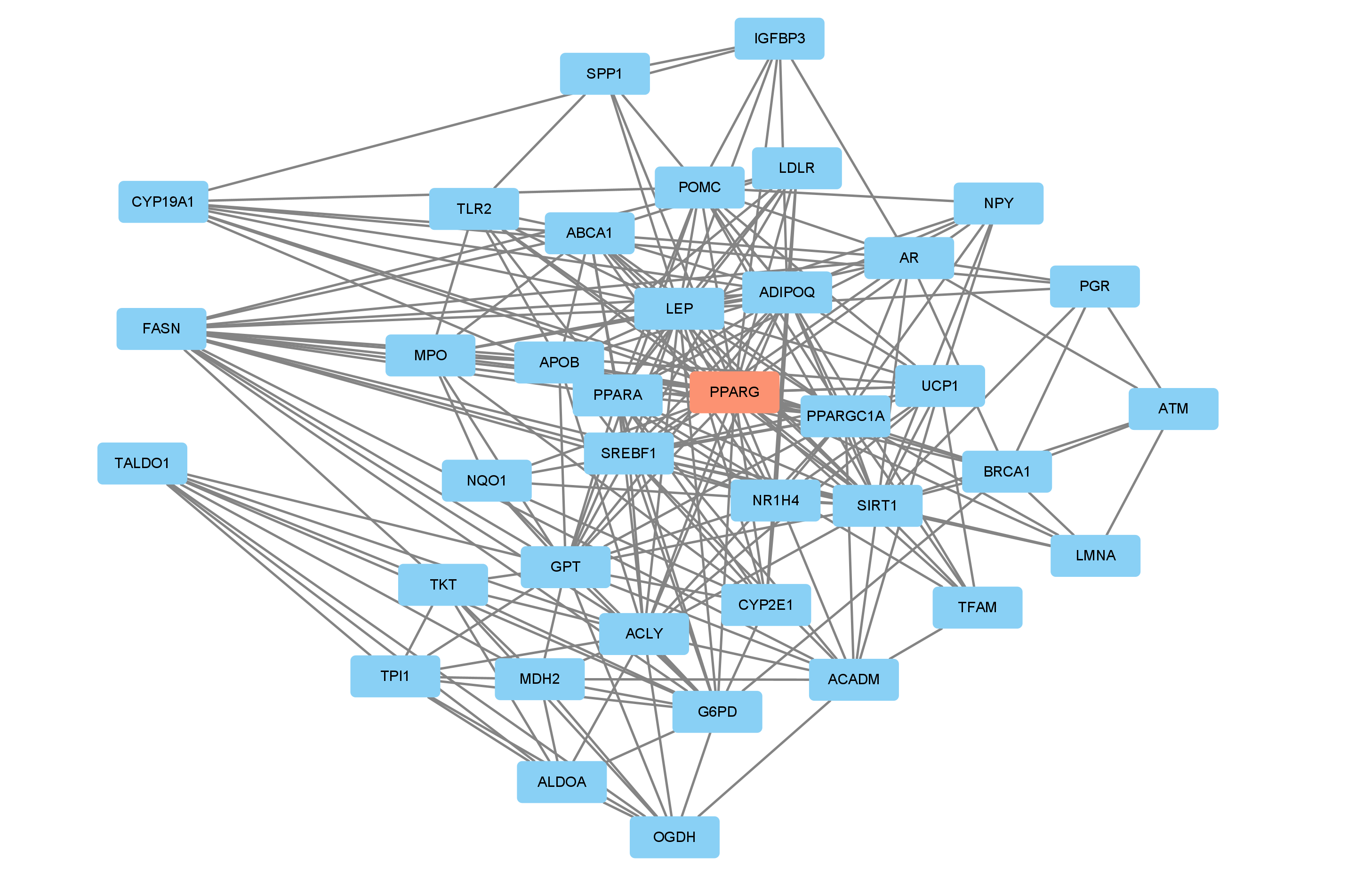

Supplement: Supplementary Figure 9 — Subnetwork and core gene PPARG most closely related to GMII gene. [file Image_9.tif]
